# Fuzzy Emotional Semantic Analysis and Automated Annotation of Scene Images

**DOI:** 10.1155/2015/971039

**Published:** 2015-03-09

**Authors:** Jianfang Cao, Lichao Chen

**Affiliations:** ^1^Department of Computer Science & Technology, Xinzhou Teachers University, Xinzhou 034000, China; ^2^School of Computer Science and Technology, Taiyuan University of Science and Technology, Taiyuan 030024, China

## Abstract

With the advances in electronic and imaging techniques, the production of digital images has rapidly increased, and the extraction and automated annotation of emotional semantics implied by images have become issues that must be urgently addressed. To better simulate human subjectivity and ambiguity for understanding scene images, the current study proposes an emotional semantic annotation method for scene images based on fuzzy set theory. A fuzzy membership degree was calculated to describe the emotional degree of a scene image and was implemented using the Adaboost algorithm and a back-propagation (BP) neural network. The automated annotation method was trained and tested using scene images from the SUN Database. The annotation results were then compared with those based on artificial annotation. Our method showed an annotation accuracy rate of 91.2% for basic emotional values and 82.4% after extended emotional values were added, which correspond to increases of 5.5% and 8.9%, respectively, compared with the results from using a single BP neural network algorithm. Furthermore, the retrieval accuracy rate based on our method reached approximately 89%. This study attempts to lay a solid foundation for the automated emotional semantic annotation of more types of images and therefore is of practical significance.

## 1. Introduction

The emotional semantic analysis of scene images is an essential component of research on high-level image semantic comprehension, pattern recognition, and computer vision. When addressing practical tasks, such as image annotation, image classification, image retrieval, face recognition, outdoor monitoring, and military reconnaissance, it is necessary to analyze human emotional behaviors in response to scene images, extract the emotional semantic features of the images, and then calculate the feature similarity degree.

The final purpose of the emotional semantic analysis of scene images is to enable computers to describe human emotional responses to scene images. Image semantics can be divided into scene, behavioral, and emotional semantics, with the last being the highest level semantics. The emotional semantic features of images are extracted based on low-level visual features: the low-level features of images, such as color, texture, shape, and contour, are first extracted using related processing technology; the correlation between the low-level features and the high-level emotional semantics is then sought to establish a mapping amongst them [1]. Emotional semantic annotation is an advanced process in the field of digital image comprehension. It is a method for automatically acquiring high-level semantics. This method provides understandable image retrieval and serves as an effective tool for the implementation of multimedia information retrieval systems. Scene images are a common type of data. Research on their semantic annotation forms the basis of the implementation of emotional semantic retrieval for other types of images and therefore has strong theoretical and practical significance.

Research on computerized emotional calculations dates back to the 1980s. Currently, reasonable approaches to analyzing emotional semantics are a popular research topic, and their development remains difficult in computational fields. To date, numerous studies have been conducted to investigate the relationship between visual image features and emotional comprehension. Yuichi and Toshikazu noted the importance of the comparisons of color and directional multiresolution for human subjective perception [2]. Mao et al. established a mathematical two-dimensional fluctuation model by analyzing the emotional features of images and proposed an analytical image fluctuation method to evaluate harmonious feelings for images [3]. Their finding indicated that images in compliance with the 1/*f* fluctuation law provoke harmonious feelings in humans. Wang et al. employed semantic quantization and factor analysis to establish an emotional space based on dimensional analysis in the field of psychology [4]. Yoshida et al. defined three emotional feelings for images, that is, comfort, disorder, and monotone [5]. Cho and Lee discussed happiness, dejection, and coolness implicated by images and conducted the semantic retrieval of images [6]. Colombo et al. defined a few commonly used adjectives, such as warm, cool, and natural, to describe the emotional semantics of images and established an emotional space [7]. Baek et al. determined 52 image patterns and 55 emotion factors that corresponded to the patterns using questionnaires and measured the relationship between low-level visual features and high-level emotion [8]. Shin et al. established an emotion prediction system to predict image emotional semantics, with an accuracy rate reaching 92% [9]. Li et al. established mapping relationships between color features and emotional semantics based on human comprehension of colors and proposed a radial basis function (RBF) neural-network-based emotional classification method for home design images based on color features [10]. According to their study, home design images can be emotionally classified into freshness, romance, coolness, and softness. Li et al. employed the fuzzy approach to describe the fuzzy semantics of artistic images and extract fuzzy semantic features to realize fuzzy semantic retrieval [11]. The accuracy rate based on their method reached approximately 70%. However, most previous research efforts focused on the mapping relationships between emotional terms and the low-level visual features of images. Studies on a probabilistic association degree between emotional terms and the semantics implicated in images are rare. In our previous study, we proposed an Adaboost-BP-neural-network-based approach to classify image semantics [12]. We used the OCC emotion model to describe image emotions and combined the outputs of 15 BP neural network weak classifiers based on the Adaboost algorithm to construct a strong classifier, which successfully increased the efficiency of emotional semantic classification. However, the following two issues were not resolved: (1) the acquisition and analysis of emotion semantic data in scene images and (2) the semantic ambiguity and subjectivity of scene images when understanding scene images, which describes the emotion degree when people understand scene images.

Subjectivity and fuzziness play important roles in human image comprehension. Finding an effective method to simulate subjectivity and fuzziness in human image comprehension will greatly enhance retrieval efficiency and lead to the realization of human-oriented image retrieval [13]. Therefore, the current study proposed a new method for extracting the fuzzy semantic features implicated by images based on fuzzy theory in combination with probability theory.

This study is organized as follows. The second section introduces the theoretical background of the emotional semantic analysis of scene images. The third section describes the experimental procedures of this study. The fourth section provides the analytical results of the experiment. The final section summarizes the main findings of this study and their implications.

## 2. Theoretical Background

### 2.1. Fuzzy Set Theory

Fuzzy set theory is an important theory in the field of artificial intelligence. It was proposed by Zadeh in 1965. He first used the term “fuzzy set” to classify fuzzy objects and used the concept of “membership degrees” to accurately describe the relationship between an element and its fuzzy set. The development of fuzzy set theory marks the birth of fuzzy mathematics [14]. Although membership degrees are the foundation of fuzzy sets, there is no one recognized standard for their determination, which is a psychological process. Currently, fuzzy theories mainly cover fuzzy set theory, fuzzy logic, fuzzy inference, and fuzzy control. The related theories and technology have matured and been widely applied.

This study employs fuzzy theory in the automated annotation of scene images. Fuzzy theory was applied to represent human emotion degrees when comprehending scene images. For example, relaxed feeling will be produced when subjects observe a scene image of nature. The degree of this type of feeling will be addressed in this study. To describe human emotions provoked by scene images, this study requires the following definitions.

1. Emotional variables: emotional variables are presented with the following five-dimensional vector: 〈*x*, *E*(*x*), *U*, *G*, *T*〉, where *x* is the name of the variables, *E*(*x*) represents the emotional value set of *x*, *U* is the domain (the extraction space of image features in this study), *G* represents the grammar laws for the generation of the emotional value of *E*(*x*), and *T* represents the semantic laws for the generation of the emotional membership degree.
2. Basic emotional value set: this refers to an emotional value set that cannot be further semantically divided.
3. Extended emotional value set: this is the emotional value set used to describe the degree of basic emotions.

### 2.2. Principal Component Analysis (PCA)

PCA was first proposed by Karl and Pearson in 1901 and is used as a tool for data analysis and mathematical model development [15]. It is a multiple statistical analysis tool that transfers multiple indices through linear combinations into several independent indices, which contain the large amount of information contained in the original indices.

The PCA algorithm is based on the following principles.

Suppose the matrix of samples can be represented as *X* = {*x*_*ij*_}_*n*×*N*_, where *n* is the feature dimensionality and *N* is the number of the samples.

(1) The sample data are standardized according to $x_{ij}^{\prime }=\left(x_{ij}-\bar{x_{i}}\right)/\sigma_{i}\;\;i=1,2,\ldots ,n$, where $\bar{x_{i}}$ is the average value of the features of the *i*th sample and *σ*_*i*_ is the standard error of the *i*th feature.

(2) Based on {*x*_*ij*_′}_*n*×*N*_, the covariance matrix *R* = {*r*_*ij*_}_*n*×*N*_ is calculated as follows:

(1) $$\begin{array}{c} r_{ij}=\frac{1}{n}\overset{n}{\underset{k=1}{\sum }}\frac{\left(x_{ki}-x_{i}\right)\left(x_{kj}-x_{j}\right)}{\sigma_{i}\sigma_{j}}. \end{array}$$

(3) Based on the feature equation |*R* − *λI*| = 0, the characteristic root *λ*_*i*_ and the eigenvector *α*_*i*_  (*i* = 1,2,…, *N*) of *R* are determined, and the characteristic roots of *λ*_*i*_ are then arranged in an ascending order as *λ*_1_ < *λ*_2_ < ⋯<*λ*_*N*_.

(4) The contribution rate *e*_*i*_ and accumulated contribution rate *E*_*m*_ of each principal component are calculated based on the following equations:

(2) $$\begin{array}{c} e_{i}=\frac{\lambda_{i}}{\sum_{k=1}^{N}\lambda_{k}},\\ E_{m}=\frac{\sum_{k=1}^{m}\lambda_{m}}{\sum_{k=1}^{N}\lambda_{k}}\;k=1,2,\ldots ,N. \end{array}$$

The contribution rate of the first principal component *e*_1_ is the proportion of the variance of the component in the total variance. A greater value indicates more information contained in the composite sample (*X*_1_, *X*_2_,…, *X*_*N*_) of the first principal component.

(5) Principal components are calculated based on the following equation:

(3) $$\begin{array}{c} F_{i}=\alpha_{1i}X_{1}+\alpha_{2i}X_{2}+\cdots +\alpha_{Ni}X_{N}\;i=1,2,\ldots ,N. \end{array}$$

## 3. Method of Fuzzy Emotional Semantic Analysis and Automatic Annotation for Scene Images

The lower-level visual features should be first extracted when images are semantically analyzed and automatically annotated. For scene images, colors are the important features that can describe emotional semantics. Therefore, this study adopted the segmentation method to extract visual features of scene images. Then, an emotional model should be established and semantic mapping should be performed to realize semantic mapping from lower-level color features to higher-level emotional semantics. Subsequently, semantic analysis and automated annotation can be performed.

### 3.1. Visual Color Feature Extraction

Color, texture, and shape are common features of images that were used. The features of the scene images were not regular, and extracting those features, for example, texture and shape, was difficult. Color was the most critical feature; it reflects the semantics of a scene image. It directly affects the human visual system and evokes different human feelings. Therefore, this study adopted the average segmentation algorithm to extract color features from images as visual features to reflect semantic information.

#### 3.1.1. Color Space Selection and Quantization

Considering that the color space of hue, saturation, and value (HSV) can satisfactorily reflect human perceptions of colors, we used HSV as the work space. Because the human visual system is more sensitive to hue than to saturation and value, we quantized the HSV spaces according to the methods used in previous studies [11, 16]. One 60-dimensional vector of color features was established to build the domain (*U*) of emotional variables. Such quantitative methods have various advantages, including better representing the human visual system, reducing color redundancy, and being applicable to gray surfaces and ease of calculation.

#### 3.1.2. Visual Feature Extraction of Colors

**Figure 1:**
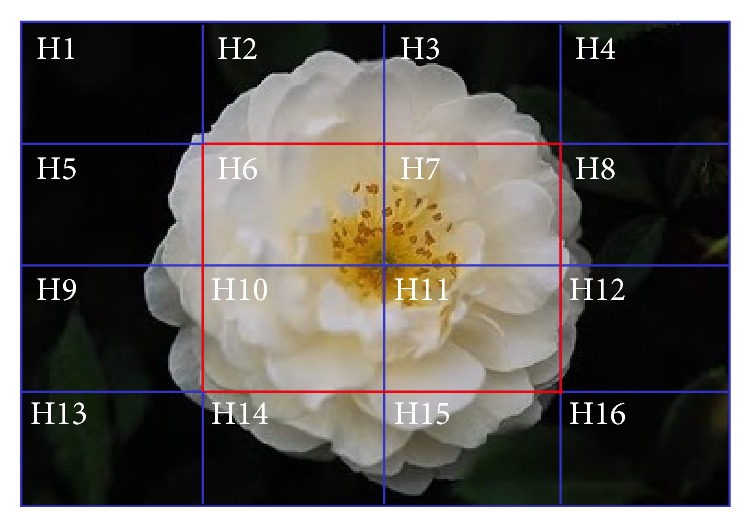
Segment layout of an image.

We adopted the deblocking strategy to extract local features of the images [17, 18]. The commonly used method is to divide images into *m* × *n* number of blocks, with different blocks having different weights. The central region of an image plays a critical role in human semantic comprehension and thus has the greatest weight. In this study, we divided the image into 4 × 4 blocks, as shown in Figure 1.

The weight of each block depends on the features of the image that is used. Because either the central block or the user-designated block normally has a large weight [18], which can better reflect the position information of the image, we increased the weight of the central blocks of the image. The weights of the blocks were distributed as follows. As shown in Figure 1, the region with red lines accounted for 1/4 of the total area of the image. We thus increased its weight to 1/3. Consequently, the weights of H6, H7, H10, and H11 were 1/12 each, and the weight of each of their surrounding 12 segments was 1/18.

After the image was deblocked, the color histogram of each block was collected. The two colors with the greatest number of pixel points in the histogram were extracted as low-level color features of the block. Then, the color features of the entire image were calculated based on the set weights.

### 3.2. Emotional Model Establishment

Emotional model establishment is critical for the emotional semantic analysis of scene images, and the determination of the basic emotional value set *E*(*x*) is a critical component in establishing the model.

Normally, the establishment of an emotional model follows three steps. First, emotional adjectives are collected to determine the emotional values. Second, a semantic quantization experiment is performed, and an emotional database is established based on the image evaluation of selected survey subjects. Third, data analysis is performed to establish the emotional space.

#### 3.2.1. The Establishment of the Emotional Value Set

Seven adjective terms were carefully selected to establish *E*(*x*) = (natural, romantic, soft, relaxed, vibrant, salubrious, changeful) as in previous studies [19]. An extended emotional value set was further constructed as {very,  neutrally,  and  hardly}. For instance, the extended emotional value set of the basic emotional value “relaxed” is {very  relaxed,  relaxed,  hardly  relaxed}.

The grammar law *G* of the emotional variables was then formulated as follows:

  〈emotion  expressional  formula〉 : : = = 〈extended  emotional  value〉∣〈basic  emotional  value〉
  〈extended  emotional  value〉 : : = = 〈membership  variables〉 & 〈basic  emotional  value〉
  〈membership  variables〉 : : = = very∣neutral∣hardly
  〈basic  emotional  value〉 : : = = natural∣romantic∣soft∣relaxed∣vibrant∣salubrious∣changeful.

Based on our experiment, three extended emotional values (very, neutral, and hardly) were quantized as follows:

(4) $$\begin{array}{c} V_{e}\left(x\right)=\left\{T_{e}^{2}\left(x\right)\;|\;x\in U\right\},\\ N_{e}\left(x\right)=\left\{\sin\left(T_{e}\left(x\right)*\pi \right)\;|\;x\in U\right\},\\ H_{e}\left(x\right)=\left\{1-T_{e}\left(x\right)\;|\;x\in U\right\}, \end{array}$$

where *e* is the basic emotional value set, *T*_*e*_(*x*) is the fuzzy membership degree obtained from the BP neural network training, *x* represents the basic emotional value, and *V*_*e*_(*x*), *N*_*e*_(*x*), and *H*_*e*_(*x*) are the membership degrees of “very,” “neutral,” and “hardly” of *x*, respectively.

#### 3.2.2. Emotional Database Establishment

The SUN Database is a free database of scene images for researchers in the field of computer image comprehension. This study adopted images from this database for the experiments [20]. A total of 100 typical scene images were selected from the SUN Database. They were used as samples for users to evaluate. These images contained different colors, cyberspace layouts, and contents. The users were 60 university freshmen, 25 females and 35 males; their ages ranged from 18 to 20. Emotional database was established based on the users' evaluation.

A total of 780 images were selected from the SUN Database and randomly divided into four groups, with 195 images in each group. The 60 users were also randomly divided into four groups, with 15 participants in each group. The users artificially annotated the selected images. An experimental platform was developed to acquire the basic emotional values of the scene images in an environment of open behaviors.

#### 3.2.3. Emotional Space Establishment and Semantic Mapping

For a sample set {(*V*_1_, *y*_1_), (*V*_2_, *y*_2_),…, (*V*_*n*_, *y*_*n*_)}, where *V*_*i*_ is the extracted 60-demensional color feature vector, *V*_*i*_ ∈ *U*, (*i* = 1,2,…, 60), and *y*_*i*_  (*i* = 1,2,…, *n*) represents the membership grade of the basic emotional value included in the extended emotion value, a mapping *T*_*e*_ : *V* → *y* *e* ∈ *E*(*x*) must be established. This mapping is the semantic law *T* of the five-dimensional emotional variables.

**Figure 2:**
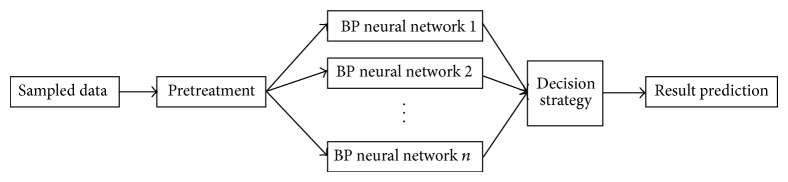
Algorithm procedure.

**Figure 3:**
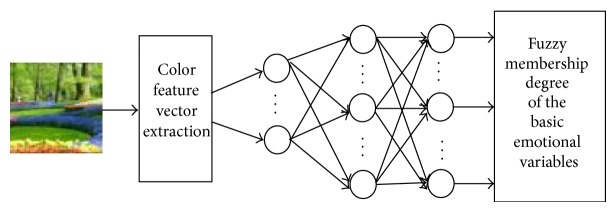
Color feature semantic extraction learning process.

Considering that the BP neural network [21] has a simple structure, a high training speed, and a strong learning capability that benefits fuzzy set processing and that the basic idea of the Adaboost algorithm is to integrate the outputs of multiple weak predicators to obtain an effective prediction, we combined the Adaboost algorithm with the BP neural network. Ten BP neural networks were repeatedly trained as weak predictors for use during output prediction; a strong predictor that was composed of the 10 weak predictors was generated based on the Adaboost algorithm. The strong predictor was then used to construct the emotional space. The flow sheet of the algorithm and the learning process of a single BP neural network are shown in Figures 2 and 3, respectively.

The BP neural network is a feed-forward neural network that is composed of an input layer, a hidden layer, and an output layer. In this study, the extracted 60-dimensional low-level visual color features were used as the network input. The element number of the hidden layer was determined to be 20. A Gaussian function was used as the activation function of the hidden layers:

(5) $$\begin{array}{c} \Phi \left(x\right)=e^{-{\left(x-\mu \right)}^{2}/\sigma^{2}}. \end{array}$$

A strong predicator was established based on the Adaboost algorithm [11]. The procedures were as follows.

(1) Data selection and neural network initialization: from the sample space, *m* groups of trained data were randomly selected. The distribution of weights of the training data was initialized according to *D*_*t*_(*i*) = 1/*m*. The neural network structure was determined according to the dimensions of the sample inputs and output. The weight value and threshold of the RBF neural network were initialized.

(2) Prediction by the weak predicator: when the *t*th weak predicator was trained, the trained data were used to train the RBF neural network, and the output was predicted. The sum of the predictive errors *e*_*t*_ of the predicted sequence *g*(*t*) was obtained based on the following equation:

(6) $$\begin{array}{c} e_{t}=\underset{i}{\sum }D_{i}\left(i\right),\;i=1,2,\ldots ,m\;\;\left(g\left(t\right)\neq y\right), \end{array}$$

where *g*(*t*) is the predicted result and *y* is the expected result.

(3) Calculation of the predicted sequence weight: according to the *e*_*t*_ of the predicted sequence *g*(*t*), the weight of sequence *a*_*t*_ was calculated based on the following equation:

(7) $$\begin{array}{c} a_{t}=\frac{1}{2}\ln\left(\frac{1-e_{t}}{e_{t}}\right). \end{array}$$

(4) Adjusting the weight of the tested data: based on the weight of the predicted sequence *a*_*t*_, the weight of the next round of trained samples was adjusted according to the following equation:

(8) $$\begin{array}{c} D_{t+1}\left(i\right)=\frac{D_{t}\left(i\right)}{B_{t}}\times \exp\left[-a_{t}y_{i}g_{t}\left(x_{i}\right)\right],\;i=1,2,\ldots ,m, \end{array}$$

where *B*_*t*_ is the normalization factor, which allows the sum of the distributional weights to be equal to 1 when the weight proportions remain unchangeable.

(5) Strong prediction function: the weights *a*_*t*_ of the *T* weak predicators were normalized after *T* rounds of trainings as follows:

(9) $$\begin{array}{c} a_{t}=\frac{a_{t}}{\sum_{t=1}^{T}a_{t}}. \end{array}$$

The prediction results of the prediction function *y*(*x*) were calculated based on the following equation:

(10) $$\begin{array}{c} y\left(x\right)=a_{t}h\left(x\right), \end{array}$$

where *h*(*x*) is the predictive value of the predicted samples obtained from the *T* weak predicators.

In the training phase, 550 images with different styles were selected from the 780 scene images and used as the training set. After training and learning, the membership degrees of the seven emotional variables were exported. For instance, for the basic emotional term “natural,” if the membership degree is 0.85, the degree of natural feeling inspired by the input image is 0.85.

Through the aforementioned procedures, image features were represented as a 7-dimensional vector; that is, *F* = {*y*_1_, *y*_2_,…, *y*_7_}, where *y*_*i*_, *i* = 1,2,…, 7, is the fuzzy membership degree of the basic emotional variables set. The corresponding basic emotional value set of the scene images in the *F* space was (natural, romantic, soft, relaxed, vibrant, salubrious, changeful).

For instance, if the semantic feature vector of a scene image after training was *F* = {0.93,0.31,0.45,0.89,0.81,0.78,0.26}, its extended emotional values are as summarized in Table 1 using (4).

### 3.3. Emotional Semantic Acquisition in an Open-Behavior Environment

We designed a website as an open experimental platform. Images from the professional psychological software E-Prime were used as demonstration images. The design of the experiment is detailed in Table 2.

Before the experiment, the test subjects were required to complete electronic documents such as a personal basic information sheet, a characteristic evaluation scale, and a feedback table. During the experiment, images were randomly displayed. Before each display, a prompt interface was shown for 1 s. After the test subjects made their choice, a rest interface was shown for 1 s to keep the subjects in a good mood. When extracted data were collected from the test subjects, the rationality and validity of the data were preliminarily evaluated. Invalid data were removed before the statistical analysis.

### 3.4. Automated Annotation of Emotional Semantics

Emotional semantic annotation attempts to realize automated image annotation by establishing a semantic conceptual model using machine learning approaches that are based on a large number of instance images. In this study, we selected the basic emotional adjective terms of fuzzy membership degrees to annotate scene images.

The membership degree of each basic emotional value corresponding to three extended emotional values is calculated. The basic emotional adjectives corresponding to the two extended emotional values with maximum membership degree plus their fuzzy membership degrees were used to annotate the images. To make the annotation easily understandable, the 7 basic emotional values corresponding to the extended emotional value “hardly” were annotated with the adjective terms holding an opposite meaning, that is, (vague, cool, hard, lifeless, desolate, narrow, tedious).

## 4. Results and Discussion

### 4.1. Emotional Semantic Data in an Open-Behavior Environment

PCA was used to collect original emotional semantic data to artificially annotate the scene images. The collected original data are partially summarized in Table 3.

**Figure 4:**
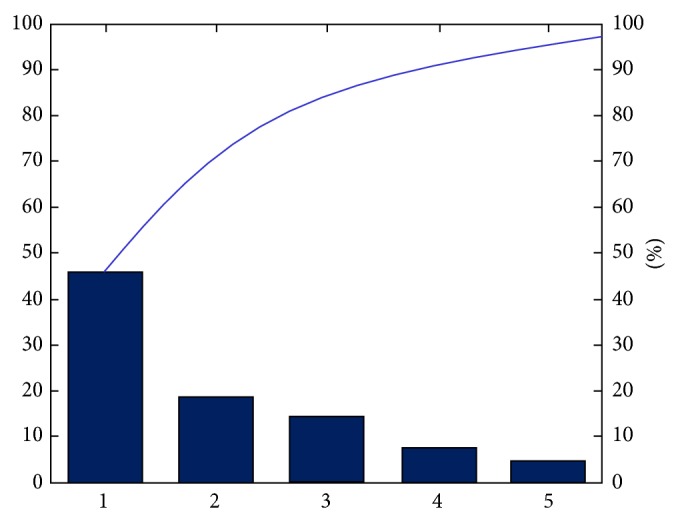
Variance contribution rates of various principal components.

The analysis results of the aforementioned data are summarized in Table 4, and the variance contribution rates of various principal components are shown in Figure 4.

Table 4 and Figure 4 show the total variance contribution rate of the first three principal components to the system reached 82%. The same method was used to analyze the selected valid data. The variance contribution rate of the three principal components in more than 90% of the samples in the system reached approximately 85%. This finding shows that the test subjects had a good attitude toward the experiments and that the collected data were valid. The first three principal components can well describe the emotional semantic information implicated in the images. The open-environment-based behavioral experiment was successful, and the acquired emotional semantic data are effective.

### 4.2. Fuzzy Emotional Semantic Automated Annotation Analysis

**Figure 5:**
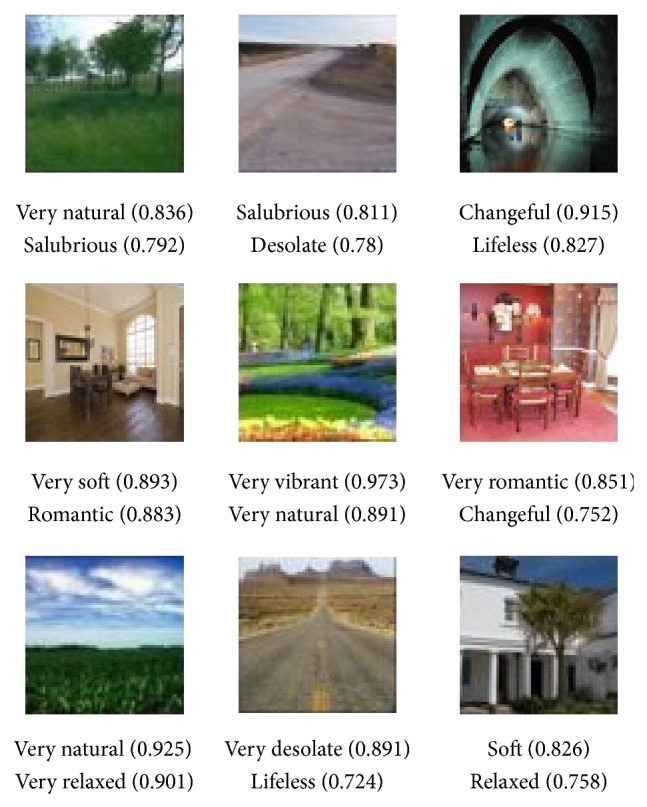
Results of automated annotation for the image emotional semantics.

In this study, 230 randomly selected scene images were tested.  Figure 5 shows the automated annotation results for the image emotional semantics.

To validate the systems' performance, the annotation results of the 230 tested images were compared with the artificial annotation results, with the latter serving as the standard. The tested users were required to provide two types of artificial annotation results: (1) those based on basic emotional values and (2) those based on extended emotional values and basic emotion values. For each image, the two adjectives that the users adopted most frequently to annotate the images were considered the artificial annotation results. The artificial annotation result does not include the membership degree. Therefore, if the automated annotation results of basic emotional value are the same as the artificial annotation results, they are considered to be correct automated annotations. First, the automated annotation results of the basic emotional values based on the weak BP neural network were compared with the artificial annotation results, with the latter serving as the standard. The BP-neural-network-based annotation accuracy rate for 197 images was 85.7% (197/230). The artificial annotation of the extended and basic emotional values was then used as the standard. The BP-based annotation accuracy rate for 169 images was 73.5% (169/230). Subsequently, the annotation results of the Adaboost algorithm-based strong predictor were compared with the artificial results. In contrast, the annotation accuracy rate of the strong predictor reached 91.2 and 82.4%. Tables 5 and 6 list the maximum, minimum, and average values of the basic and extended emotional values using the artificial and automatic annotation approaches.

Two types of models were used in this test. The results in Table 5 do not include the annotation results of the basic emotional values with fuzzy membership; thus, the systemic annotation accuracy was higher.  Table 6 lists the systemic annotation results after the extended emotional values were added; the annotation accuracy was comparatively lower, which leads to the conclusion that different users have essentially identical emotional comprehension for the same image. However, the differences in their degrees of comprehension vary too much for a single conclusion to be reached. The Adaboost algorithm and the BP neural network are combined to increase the systemic annotation accuracy to demonstrate the advantages of the Adaboost algorithm. Furthermore, for emotional adjective terms such as romantic and changeful, the annotation accuracy is too low and thus shows that the related visual elements are fuzzy from an emotional comprehension perspective. Li et al. employed a fuzzy method to extract fuzzy semantic features of art scene images to analyze and retrieve the fuzzy semantics of such images [22]. They selected an artificial neural network to produce the semantic mapping, and the average accuracy reached approximately 68%. In this study, we employed fuzzy theory and the automated annotation while adopting the Adaboost-BP neural network to produce semantic mappings. When extended emotional values were considered, the annotation accuracy was greater than 86%. Even when extended emotional values were considered, the annotation accuracy was greater than 74%. These results may have been due to a number of reasons. On one hand, art images are more abstract and include more complicated underlying emotional semantics. On the other hand, the Adaboost-BP neural network, which was used in this study, exhibits a much higher accuracy than other artificial algorithms. Therefore, this method applied fuzzy theory to the automated annotation of scene images to obtain a better performance and determine human emotional degrees when analyzing scene images.

**Figure 6:**
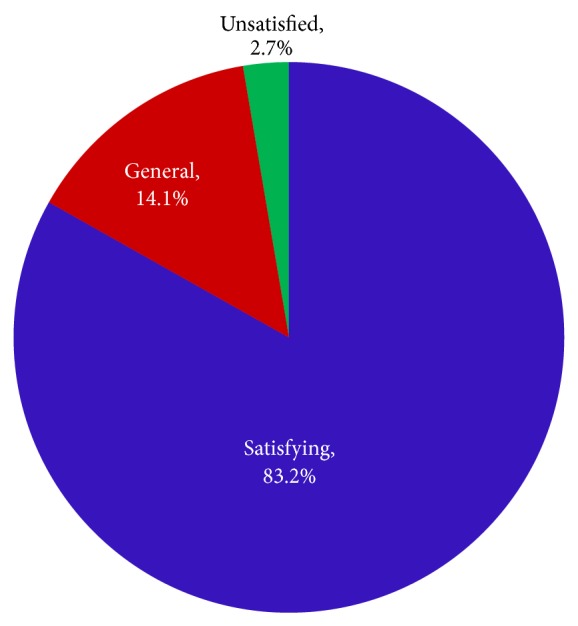
Satisfaction survey statistics.

To better demonstrate the validity of the fuzzy-theory-based annotation approach proposed in this study, satisfaction surveys were created to allow the users to evaluate the emotional semantic annotation of the scene images on our website. Of these satisfaction surveys, 509 are considered as valid (Figure 6).

Figure 6 shows that 97.3% of the tested users were satisfied with the annotated results. This finding shows that the approach proposed in this study is clearly valid.

In addition, to validate the effectiveness of the proposed approach, this study performed automated annotation and retrieval on 2000 randomly selected images. The retrieval accuracy for this process reached 89.3%. Li et al. employed a fuzzy approach to extract fuzzy semantic features in artistic images. They attempted to address the human subjectivity and fuzziness of retrieval and adopted the mapping method for neural networks. As the retrieval results of their experiment using 5000 images show, the retrieval accuracy reached approximately 70% [11]. Compared with the approach proposed by Li et al., the retrieval accuracy based on our method increased by 19%. The resulting accuracy is presumably the result of the following. On one hand, this study involved the establishment of a strong predicator with the Adaboost algorithm to greatly increase its efficiency. On the other hand, scene images are not as abstract as artistic images; therefore, the human subjects could more easily understand and classify them.

The automated annotation based on emotional semantics is a branch of image annotation and information retrieval. The main purpose of this field is to empower computers to automatically analyze the emotional semantics implied in images. Currently, scholars in the field of image retrieval and computer vision are actively performing various studies. Hayashi and Hagiwara used color histograms as image emotional features to establish the mapping relationship between colors and emotions using [23]. Dai analyzed the texture parameters of the grayscale coexisting matrix of the HSV color component and determined the influence of textures on five types of emotions [24]. Colombo et al. borrowed from Johannes' theory of semantics implied by lines, colors, and shapes to realize the inference from low-level visual features to high-level image representation features and then established a mapping reasoning mechanism [25]. Boato et al. used a support vector machine as the classifier to recognize the emotions provoked by natural images and achieved good results [26]. However, these studies mainly focused on the exploration of appropriate machine learning and advanced algorithms of artificial intelligence to realize automated computer annotation of image emotions [27–29]. In contrast to the abovementioned studies, this study attempted to explain the semantics of image from the perspective of cognition and deblocked the components of images. For the image emotion model, the current study directly borrowed from the results of psychology and established a more scientific framework for image emotion description. For image annotation, this study combined automated annotation with manual annotation, which has been demonstrated to be an effective method for the enhancement of annotation accuracy based on the results of this study. However, studies on emotional image comprehension remain in their infancy. The quantization scale of scene images needs to be further investigated. Furthermore, the development of more realistic annotation technology is necessary. Establishing the retrieval system based on emotions is a topic that needs to be addressed.

## 5. Conclusions

A serious challenge in the field of artificial intelligence is discovering how computers can mimic human perception and expression to realize human-machine harmony. In this study, we employed fuzzy set theory to explore the relationship between scene images and human subjective comprehension. The model was established based on the Adaboost algorithm and a BP neural network to realize the automated annotation of a scene image's emotional semantics. The findings proved that the combination of the BP neural network and the Adaboost algorithm exhibits more advantages when they are applied to address human subjectivity and the uncertainty of emotional subjects. The study not only proposes one approach to addressing human subjectivity and fuzziness but also provides new insights to understanding other types of image emotional semantics.

However, this study has the following limitations. First, all of the tested users were from the same source; therefore, errors may be present in our findings. Increasing the number of tested users may help the research results be more accurate and objective. Second, the artificial neural network model requires more training data. If the experiment used a larger training data set, its result would be more accurate. Nevertheless, the algorithm would require excessive computational time and human resources. Therefore, obtaining higher annotation accuracy with a small group of samples will be the focus of further research. Third, the way to standardize the classification of image emotion semantics must be further investigated because scene images possess rich semantic content.

## Figures and Tables

**Table 1:** Extended emotional values of image semantic features.

Basic emotion value	Semantic feature value	Membership degree of extension emotion value		
		Very	Neutral	Hardly
Natural	0.93	0.865	0.220	0.07
Romantic	0.31	0.096	0.827	0.69
Soft	0.45	0.203	0.988	0.55
Relaxed	0.89	0.792	0.340	0.11
Vibrant	0.81	0.656	0.563	0.19
Salubrious	0.78	0.608	0.638	0.22
Changeful	0.28	0.078	0.770	0.72

**Table 2:** Experimental design of scene image emotional semantic annotation.

Emotion classification	Annotation value	Annotation method	Image show time
Natural	0	Click the left mouse key; one image annotates one emotional classification	6 seconds
Romantic	1		
Soft	2		
Relaxed	3		
Vibrant	4		
Salubrious	5		
Changeful	6		

**Table 3:** Portion of the original testing data.

Test subject	s1.jpg	s2.jpg	s3.jpg	s4.jpg	s5.jpg	s6.jpg	s7.jpg	s8.jpg
1	0	5	1	5	6	3	4	1
2	0	2	6	0	5	4	0	1
3	3	3	1	4	6	5	4	6
4	4	5	6	4	3	3	4	3
5	3	3	6	5	6	3	0	1
6	3	2	2	4	6	4	0	1
7	0	3	6	0	5	3	0	3
8	0	3	2	5	6	5	4	6
9	4	2	1	5	3	3	4	3
10	3	5	1	5	5	3	4	1

**Table 4:** Rules of analysis of emotional semantic data for scene images.

Principal component	Characteristic value	Variance contribution rate	Accumulated contribution rate
1	11.7990	47.6836	47.6836
2	4.8186	19.4736	67.1572
3	3.8096	15.3956	82.5528
4	1.9662	7.9459	90.4986
5	1.4807	5.9841	96.4827
6	0.6134	2.4791	98.9618
7	0.1855	0.7496	99.7114
8	0.0714	0.2886	100.0000

**Table 5:** Annotation accuracy of seven types of basic emotional values (%).

User annotation	Model	Natural	Romantic	Soft	Relaxed	Vibrant	Salubrious	Changeful
Maximum value	BP	93.2	85.7	84.1	85.7	90.6	91.4	83.5
	Adaboost-BP	95.4	88.5	87.8	89.7	93.6	93.9	85.1

Minimum value	BP	87.1	77.6	80.2	78.2	86.1	85.9	76.9
	Adaboost-BP	89.8	80.1	83.7	82.3	89.4	89.3	78.2

Average value	BP	91.4	82.0	81.7	83.8	88.4	87.8	81.9
	Adaboost-BP	93.2	84.6	84.0	86.9	90.5	91.7	83.6

**Table 6:** Annotation accuracy of extended emotional value (very) with seven basic emotional values %.

User annotation	Model	Natural	Romantic	Soft	Relaxed	Vibrant	Salubrious	Changeful
Maximum value	BP	83.7	73.6	78.0	82.7	80.4	84.4	71.8
	Adaboost-BP	90.3	80.1	83.4	85.9	87.4	89.2	76.9

Minimum value	BP	76.1	68.8	70.2	74.2	71.9	74.9	64.3
	Adaboost-BP	81.8	72.1	76.1	80.1	78.5	80.3	72.0

Average value	BP	79.2	70.6	73.4	78.4	75.8	78.1	68.3
	Adaboost-BP	85.2	75.5	79.2	82.5	83.1	84.8	74.1
